# Radical Scavenging and Antimicrobial Properties of Polyphenol Rich Waste Wood Extracts

**DOI:** 10.3390/foods9030319

**Published:** 2020-03-10

**Authors:** Anita Smailagić, Petar Ristivojević, Ivica Dimkić, Tamara Pavlović, Dragana Dabić Zagorac, Sonja Veljović, Milica Fotirić Akšić, Mekjell Meland, Maja Natić

**Affiliations:** 1 Innovation Center of the Faculty of Chemistry, University of Belgrade, P.O. Box 51, 11158 Belgrade, Serbia; anitasmailagic@yahoo.com (A.S.); naca10@gmail.com (D.D.Z.); 2 Faculty of Chemistry, University of Belgrade, P.O. Box 51, 11158 Belgrade, Serbia; ristivojevic@chem.bg.ac.rs; 3 Faculty of Biology, University of Belgrade, Studentski trg 16, 11000 Belgrade, Serbia; ivicad@bio.bg.ac.rs (I.D.); tamara.pavlovic@bio.bg.ac.rs (T.P.); 4 Institute of General and Physical Chemistry, University of Belgrade P.O. Box 551, 11001 Belgrade, Serbia; pecic84@hotmail.com; 5 Faculty of Agriculture, University of Belgrade, 11080 Belgrade, Serbia; fotiric@agrif.bg.ac.rs; 6 Norwegian Institute of Bioeconomy Research-NIBIO Ullensvang, NO-5781 Lofthus, Norway; mekjell.meland@nibio.no

**Keywords:** wood waste, phenolic profile, planar chromatography, DPPH-HPTLC assay, antimicrobial activity

## Abstract

The main focus of this study is to assess radical scavenging and antimicrobial activities of the 11 wood extracts: oak (*Quercus petraea* (Matt.) Liebl*., Q*. *robur* L., and *Q. cerris* L.), mulberry (*Morus* *alba* L.), myrobalan plum (*Prunus* *cerasifera* Ehrh.), black locust (*Robinia pseudoacacia* L.), and wild cherry (*Prunus avium* L.). High-performance thin-layer chromatography (HPTLC) provided initial phenolic screening and revealed different chemical patterns among investigated wood extracts. To identify individual compounds with radical scavenging activity DPPH-HPTLC, assay was applied. Gallic acid, ferulic and/or caffeic acids were identified as the compounds with the highest contribution of total radical scavenging activity. Principal component analysis was applied on the data set obtained from HPTLC chromatogram to classify samples based on chemical fingerprints: *Quercus* spp. formed separate clusters from the other wood samples. The wood extracts were evaluated for their antimicrobial activity against eight representative human and opportunistic pathogens. The lowest minimum inhibitory concentration (MIC) was recorded against *Staphylococcus aureus* for black locust, cherry and mulberry wood extracts. This work provided simple, low-cost and high-throughput screening of phenolic compounds and assessments of the radical scavenging properties of selected individual metabolites from natural matrix that contributed to scavenge free radicals.

## 1. Introduction

Ageing processes of some alcoholic beverages are one of the most important practices during their production. This contributes to improved sensory characteristics such as aroma, color, taste and astringency. The most commonly used material in cooperage is oak heartwood barrels. Alternative wood species such as chestnut, cherry and mulberry are also used in Balkan cooperages, in different forms such as wood chips and staves [1]. Nowadays, notable studies have showed that agri-food wastes and by-products, including waste from barrel production, represent an inexhaustible source of valuable biologically active compounds. Additionally, this waste represents a low-cost material, which can be used as material for the production of the extracts. Recently, various extraction techniques were reviewed and compared with classical extraction procedures used for recovery of the antioxidant compounds from wastes [2]. Using simple, fast and inexpensive eco-friendly extraction methods for phenolic compounds represents an efficient method and advantage for further implementation in the food, pharmaceutical and cosmetic industries [3,4,5]. 

From the production of wood barrels, it is estimated that more than 200 tons of wood waste is available annually in Serbia [6]. Forests and other wooded land occupy ~2.5 million hectares, which is about one third of the territory of the Republic of Serbia. These natural populations of Serbia contain a large number of economically important forest tree species (oak, beech, black locust, spruce, pine and fir) together with autochthonous and introduced wild fruit trees species (wild cherry, cherry plum, mulberry, wild pear, wild apple, cornelian cherry, hazel and walnut) which are used for timber production, afforestation and erosion prevention, for grafting, in human diet, in medicine, in industrial processing and in landscape architecture [7,8].

In Serbia, barrels are mostly made of oak (pedunculate oak, *Quercus robur,* or sessile oak, *Quercus petraea* (Matt.) Liebl. L.) and Turkey oak (*Quercus cerris* L.) but sometimes black locust (*Robinia pseudoacacia* L.), myrobalan plum (*Prunus cerasifera* Ehrh.), mulberry (*Morus alba* L.) and even wild cherry (*Prunus avium* L.) are used as a cheaper substitute. Oak is the most widespread deciduous tree in Serbia, a national tree with strong historical and religious importance. Myrobalan plum is a native tree in Southeast Europe, has great genetic importance for horticultural breeding, and has spread throughout the whole country in all kinds of micro-climatic and pedologic conditions. Mulberries are very common, since ex-Yugoslavia used to be the fifth largest silk producer in the world with more than 2.5 million white mulberry trees [9]. Wild cherry, a noble tree, is widely distributed by birds; its seeds are used for generative rootstock production and its fruits are suitable for table consumption and as a local medicine [10,11]. Black locust is mostly used in construction works, as technical or ornamental wood, and is most commonly used as firewood. It has special value as a honey species for beekeeping [12].

Wood waste has a potential to be reused in the food and pharmaceutical industry due to its richness in potentially bioactive phenolic compounds with high antioxidant and antimicrobial activity. In our previous research [6], ellagic acid was abundant in sessile and pedunculate oak wood. It was also found in Turkey oak, black locust and myrobalan plum, but in much lower quantities. Mulberry contained the largest concentration of *p*-hydroxybenzoic acid and stilbenoids in comparison with other wood species, while myrobalan plum showed the highest content of protocatechuic acid and 5-*O*-caffeoylquinic acid. Wild cherry was characterized by richness in flavonols, flavanones, flavones, isoflavones and flavanonols [6,13,14], with taxifolin as the most abundant phenolic compound [6]. Extracts from sessile and pedunculate oak, black locust, myrobalan plum, wild cherry and mulberry showed notable antioxidant capacity, with the highest radical scavenging activity in the latter extract. Turkey oak showed the lowest radical scavenging activity [6]. According to the literature, phenolic acids were identified as the major contributors to the antioxidant capacity in wood samples, including gallic, protocatechuic, *p*-coumaric and ellagic acid and all the ellagitannins, due to their characteristic structure [15]. The following phenolic acids: ferulic acid, caffeic acid, protocatechuic acid, gallic acid, *p*-coumaric acid and chlorogenic acid, also present in some wood species, exhibit strong free radical scavenging properties on silica plates [16].

It is proposed that phenolic compounds can damage the bacteria cell membrane by interacting with the proteins of the cell membrane, or can be involved in interaction with cellular enzymes [17], which may directly or indirectly cause metabolic dysfunction and finally bacterial death [18]. Phenolic compounds are able to inhibit bacterial quorum sensing signal receptors, enzymes and secretion of toxins [19]. The type, structure and concentration of phenolic compounds, as well as the microorganism used, will influence the bacterial growth. Large doses of phenolic compounds may be toxic for bacteria, but lower doses can be used as substrates [17]. 

Some phenolic compounds present in several wood species showed antimicrobial activity. Taxifolin exhibited antibacterial activity against six known clinical pathogens: *Escherichia coli*, *Listeria* sp., *Pseudomonas aeruginosa*, *Bacillus* sp., and *S. aureus* [20]. Oxyresveratrol, the most abundant stilbene in mulberry, was active against the methicillin-resistant *S. aureus* [21].

Among flavonoids present in wild cherry wood, flavonols were distinguished by effective antimicrobial activity against resistant bacteria [22]. Methanolic extract (80%, *v*/*v*) from oak bark (*Q. robur* L.) showed moderate bactericidal, fungicidal, bacteriostatic and fungistatic activity on *S. aureus*, *Enterobacter aerogenes* (today known as *Klebsiella aerogenes*) and *C. albicans* [23]. *Q. robur* bark showed strong antibacterial activities against *Pseudomonas aeruginosa, M. flavus* and *E. coli,* and moderate effects against other bacterial species [24]. Heartwood and resin of cherry wood exhibited cytochrome inhibition and antifungal activity [25], while cherry wood extracts possessed noticeable antimicrobial activity against 9 out of the 11 wine organisms tested [17]. Oak wood has abundant ellagitannins, which are toxic to microorganisms, and provides good resistance to fungal degradations [26]. 

Antimicrobial resistance presents a global problem since resistant pathogens can cause life-threatening conditions that become incurable with one or more known drugs. The mechanisms of the antibacterial activities of many plant-derived flavonoids are different than those of conventional drugs, which open new possibilities in enhancement of antibacterial therapy [27]. In addition, many synthetized drugs have side-effects, which are small in the case of plant-derived compounds [27]. Due to all these reasons, the development of alternative drugs derived from natural resources is an attractive option.

Radical scavenging activity using DPPH-HPTLC (high performance still layer chromatography) assay and antimicrobial activity on wood waste extracts are not investigated so far. Thus, the main aim of this research was to assess radical scavenging and antimicrobial activities of the wood waste extracts from mulberry (*M. alba* L.), myrobalan plum (*P. cerasifera* Ehrh.), black locust (*R. pseudoacacia* L.), wild cherry (*P. avium* L.), and different species of oaks (*Q. petraea* (Matt.) Liebl*., Q*. *robur* L. and *Q. cerris* L.) and consider their usage in the pharmaceutical and food industries. Phenolic compounds were separated by using HPTLC, while radical scavenging activity was determined using DPPH-HPTLC. 

## 2. Materials and Methods

### 2.1. Chemicals

Ethyl acetate was purchased from Merck (KGaA, Darmstadt, Germany); formic acid, hexan 2,2-diphenyl-1-picrylhydrazyl (DPPH) and phenolic standards from Sigma-Aldrich (Steinheim, Germany); and 2-aminoethyl diphenylborinate (NTS) from Fluka (Steinheim, Germany). Gallic acid, ferulic acid and caffeic acid were supplied by Sigma Aldrich (Steinheim, Germany). 

### 2.2. Samples and Preparation of Wood Extracts

Eleven different wood staves of different geographical origins were analyzed (Table 1). In total three samples of Pedunculate oaks (*Quercus robur* L.), three of sessile oaks (*Quercus petraea* (Matt.) Liebl), and one sample of Turkey oak (*Quercus cerris* L.), black locust (*Robinia pseudoacacia* L.), myrobalan plum (*Prunus cerasifera* Ehrh.), wild cherry (*Prunus avium* L.), and mulberry (*Morus alba* L.) were included. Nine staves were stored for the whole year in the open air at cooperage industry VBX-SRL. D.O.O. in Kraljevo, Central Serbia., while two samples (sessile oak from Kuršumlija and Turkey oak) were not seasoned [6]. The wood age of the oak wood staves was over 60 years, while the wood age of non-oak wood staves was more than 40 years.

Firstly, the staves were grinded in a mill for wood and sieved until granulation of 0.5–1.5 mm was obtained. The sawdust (2.5 g) was extracted with 25 mL of ethanol (60%, *v/v*), in Erlenmeyer flasks, with constant stirring in a magnetic stirrer for seven days in darkness and room temperature (20 ± 2 °C) [6]. The extracts were centrifuged twice (5 min at 8000 rpm). For investigation of antimicrobial activity, the extracts were evaporated with a rotary evaporator and diluted in methanol until the concentration of 50 mg mL^−1^ was reached. The extraction yield of each extract was calculated from the weight of the extract residue obtained after solvent removal and the weight of waste wood employed in the extraction procedure.

### 2.3. High-Performance Thin-Layer Chromatography and Image Analysis

HPTLC Silica gel 60F_254_ plates were used for both HPTLC fingerprint and DPPH-HPTLC assay (Merck, Germany). The oak and wild cherry samples (5 μL), black locust, myrobalan plum and mulberry (2 μL), and four standard compounds: gallic acid, ferulic acid, caffeic acid and *p*-coumaric acid (2 μL, c = 1000 ppm), were applied as bands (8 mm) using Linomat 5 system (Camag, Muttenz, Switzerland). 

The mobile phase consisted of a mixture of ethyl acetate:hexan:formic acid:water (11:2:1:0.5 *v*/*v*/*v*/*v*). The plates were developed at room temperature (20 °C) in a twin-trough-chamber (CAMAG) saturated with the vapors of mobile phase for 15 min, at a developing distance of 70 mm. The obtained HPTLC chromatograms were derivatized with 2-aminoethyldiphenylborate solution (NTS - 0.2% in ethanol) in order to intensify the fluorescence of compounds. 

For DPPH-HPTLC assay, a developed HPTLC chromatogram was immersed manually for 3 seconds (s) in DPPH**·**methanol solution (0.2%) and then photographed every 30 s for 15 min. Images of the plates were captured with mobile phone (Huawei P Smart) equipped with a 13-pixels camera. All developed plates were photographed both before and after derivatization and saved as TIF files.

Images of the HPTLC chromatograms were analyzed using free available Image J software. The obtained results for each sample were cropped and denoised by using median filter with three pixels width filter. Further, images were transformed and the tracks were outlined with a rectangular selection tool. The line profile plots were generated with Plot Profile option for each sample. Profile plot displays a 2-D graph of the intensities of pixels along a line.

### 2.4. Principal Component Analysis

The line profiles were obtained using ImageJ software [28]. Principal Component Analysis (PCA) was applied using PLS ToolBox, v.6.2.1 (Eigenvector Research, Inc. 196 Hyacinth Road Manson, WA 98831, USA), for MATLAB (7.12.0(R2011a) (http://www.eigenvector.com/software/pls_toolbox.htm, Eigenvector Research, Inc., Wenatchee, WA). The data were pre-processed using correlated optimized warping (COW), standard normal variate (SNV) and mean centering to improve multivariate models.

### 2.5. Bacterial Strains and Growth Conditions

Antibacterial activity was tested using eight indicator strains in line with their growth requirements (Table 2). Suspensions were adjusted to McFarland standard turbidity (0.5) (BioMérieux, Marcy-l’Étoile, France), which corresponds approximately to 1 × 10^8^ CFU mL^−1^.

### 2.6. Well-Diffusion Method

A modified well-diffusion method [30] was performed for initial screening of the antimicrobial potential of the selected 11 wood waste extracts. Wells were made of sterile bottom parts of pipette tips (200 µL) and placed on the LA/BHA/TSA solid medium (Table 2). According to growth requirements of used strains, 6 mL of LA/BHA/TSA soft agar was inoculated with 60 µL of the appropriate strain and poured into Petri dishes over the solid medium. Molds (5 mm in diameter) were removed after soft agars solidification and 20 µL of each extract (1 mg/well) was added. Vancomycin and nystatin were used as a positive control (antibiotic/mycotic, viz., 0.2 mg/well), for bacterial strains and *C. albicans*, respectively. As a negative control, 20 μL of methanol was used. The Petri dishes were incubated at 37 °C, for 24 h. After the incubation, bacterial susceptibility and zones of inhibition were measured and expressed in mm.

### 2.7. MIC Assay

A broth microdilution method was used to determine the minimum inhibitory concentration (MIC) and minimum bactericidal concentrations (MBC) of the selected 11 wood waste extracts. Extracts were tested in the concentration range from 0.02 to 2 mg mL^−1^ by performing two-fold serial dilutions with the appropriate medium in 96-well microtiter plates. Negative control (control of bacterial and yeast growth) and sterility control (blank, only appropriate medium) were also tested. The final concentration of the solvent control (methanol) in the first wells was 10%. Vancomycin, streptomycin and nystatin were tested as positive controls in concentration range from 0.001 to 0.4 mg mL^−1^. Beside negative and sterility controls, each well was inoculated with 20 µL of bacterial/yeast culture (approx. 1 × 10^6^ CFU mL^−1^), reaching a final volume of 200 μL. In addition, 22 µL of resazurin indicator was added to each well. Microtiter plates were incubated for 24 h at 37 °C. In the presence of living bacterial cells, blue colored resazurin was being irreversibly reduced to pink colored and highly red fluorescent resorufin [31]. The lowest concentration of each extract which showed no change in color of resazurin was defined as MIC value. MBC/MFC values were determined by sub-culturing the dilutions from wells without color changes on agar plates. Plates were incubated 24 h at 37 °C and bacterial/yeast growth was monitored. The lowest concentration without growth was defined as MBC/MFC value. The results were expressed in mg mL^−1^.

## 3. Results and Discussion

### 3.1. Line Profiles of Investigated Extracts

**Figure 1:**
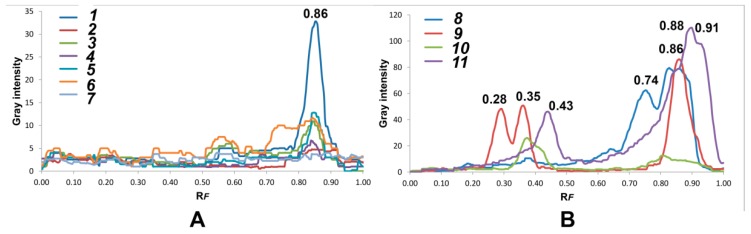
Line profiles of investigated wood extracts based on HPTLC analysis: (**A**) oak samples (no. 1–7); (**B**) non-oak samples (no. 8–11): black locust (*Robinia pseudoacacia* L.) (no. 8), myrobalan plum (*Prunus cerasifera* Ehrh.) (no. 9), wild cherry (*Prunus avium* L.) (no. 10) and mulberry (*Morus alba* L.) (no 11).

**Figure 2:**
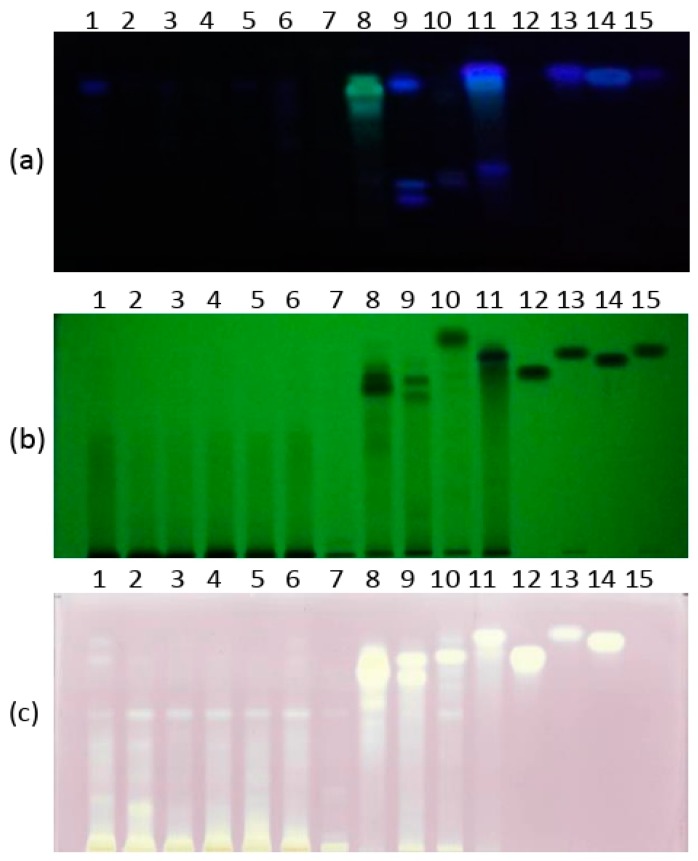
HPTLC chromatograms of samples: *Q. robur* (no. 1–3), *Q. petraea* (no. 4–6), *Q. cerris* (no. 7), *Robinia pseudoacacia* (no. 8), *Prunus cerasifera* (no. 9), *Prunus avium* (no. 10), mulberry (no. 11) and four standard compounds (gallic acid (no. 12), ferulic acid (no. 13), caffeic acid (no. 14) and *p*-coumaric acid(no. 15)); (**a**) under UV light at 366 nm; (**b**) under UV light at 254 nm; (**c**) DPPH-HPTLC chromatogram.

Investigated wood samples contained several characteristic phenolic compounds at R*_F_* values of: 0.28, 0.35, 0.43, 0.74, 0.86 (gallic acid), 0.91 (ferulic acid), 0.88 (caffeic acid) and 0.91 (*p*-coumaric acid) (Figure 1). Based on HPTLC profiles, wood extracts contained bands with R*_F_* values from 0.28 to 0.91. There are five different patterns in the investigated samples: *Quercus* samples showed one band of weak intensity with R*_F_* value at 0.85, while sample 1 (Pedunculate oak—*Q. robur* L.) had the highest intensity peak of this compound. Further, black locust (sample 8) showed greenish bands with R*_F_* at 0.75, 0.82 and 0.87, clearly different from standard phenolic acids (Figure 2a).

Wild cherry contained one characteristic peak at 0.36, whereas myrobalan plum had a different pattern from other wood samples with three characteristic peaks at R*_F_* values of 0.28, 0.35 and caffeic acid (Figure 1). A different profile of myrobalan plum in comparison with other wood samples could be seen also by HPLC [6], where it contained significantly larger amounts of protocatechuic acid and 5-O-caffeoylquinic acid than other wood samples. The peak profiles for mulberry showed it contained peaks at 0.43, 0.84, gallic, ferulic and/or *p*-coumaric acids. 

Mulberry sample showed hardly visible blue band with R*_F_* at 0.86, recognized as gallic acid, while black locust and oak wood samples contained gallic acid in higher amounts. In addition, wild cherry contained *p*-coumaric and ferulic acid in greater quantities than mulberry, and caffeic acid in greater quantities than myrobalan plum, which was observed neither on HPTLC plates nor line profiles. 

### 3.2. DPPH-HPTLC Assay

It was previously shown that the total antioxidant activity of each extract through the DPPH assay, mulberry and myrobalan plum wood extracts had significantly higher DPPH values in comparison to the other samples [6]. The single compounds with radical scavenging activity and their contribution to the total radical scavenging activity were investigated by DPPH˙-HPTLC assay. Substances exhibiting radical scavenging properties (yellow bands against a purple background) were located between R*_F_* values at 75 and 92 (Figure 2c). The most dominant zones in the HPTLC-DPPH˙ fingerprints were compounds with R*_F_* values at 0.87, 0.91 and 0.92, which could be recognized as gallic, ferulic and/or caffeic acids (zone 4). Extracts no. 8–11 showed strong radical scavenging activities, mainly due to the previously detected phenolic compounds, while *Quercus* samples revealed one weak band with R*_F_* at 75 against the purple background. *P. cerasifera* contained two bands at 0.84 and caffeic acid, and were recognized as radical scavengers. The, *M. alba* sample showed radical scavengers with R*_F_* values at 0.84, gallic, ferulic and/or *p*-coumaric acids. These compounds have been recognized before as strong radical scavengers on silica plates [16]. 

### 3.3. Principal Component Analysis

Visual inspection of HPTLC chromatograms is a subjective method and mainly depends on the analyst’s perception. On the other hand, multivariate chemometrics analysis applied on the HPTLC chromatogram provides an objective classification of the investigated samples for identification of phenols most responsible for classification, as well as identification of outliers. The HPTLC system was optimized to separate and identify all phenols from different wood extracts.

**Figure 3:**
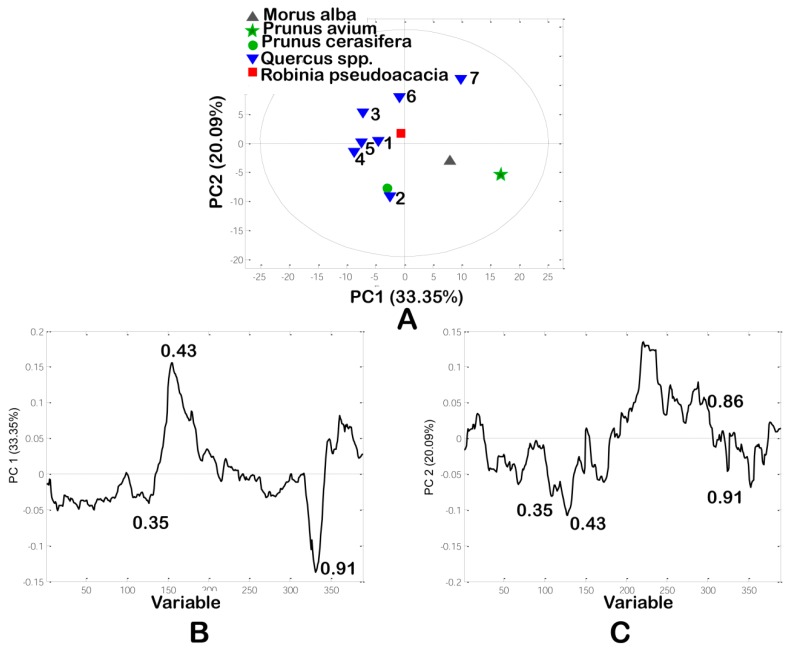
Principal component analysis (PCA) of HPTLC chromatogram: (**A**) The PC score plot; (**B**) and (**C**) The loading plots. 1–3 Pedunculate oaks (*Quercus robur* L.), 4–6—sessile oaks (*Quercus petraea* (Matt.) Liebl), 7—Turkey oak (*Quercus cerris* L.).

Principal component analysis (PCA) is a commonly used multivariate technique. It accounts for most of the variation of total variability, visualizes the structure of data by grouping objects into two or three dimensions, and identifies important variables responsible for discrimination between wood samples. PCA as an initial multivariate technique was applied on the data matrix (11 samples × 389 variables) obtained from HPTLC chromatograms, where variables represent the intensities of pixels along the 389 length lines. The first two Principal Components (PCs) accounted for 33.35% and 20.09% of the total variability, respectively. The first five principal components describe 87.85% of total variability. From the PC score plot (Figure 3a), six *Quercus* samples were positioned on left side of PC score plot, while other four wood samples were misclassified and positioned on right side of PCs score plot. The loading plots (Figure 3b,c) demonstrated the significant contribution of polyphenolic compounds to the total variability. The most influential phenolic compounds discriminating between *Quercus* and the other wood samples were compounds with R*_F_* values at 0.35, 0.43, 0.86 and 0.91. In contrast to other types of wood samples *Quercus* samples contained low amounts of phenolic compounds with R*_F_* values at 0.35, 0.43, 0.86 and 0.91. Polar compounds with low R*_F_* values could be some phenolic acids and/or glycosides. These phenolic compounds may be identified as characteristic taxonomical markers between wood species.

### 3.4. Well-Diffusion Method

**Figure 4:**
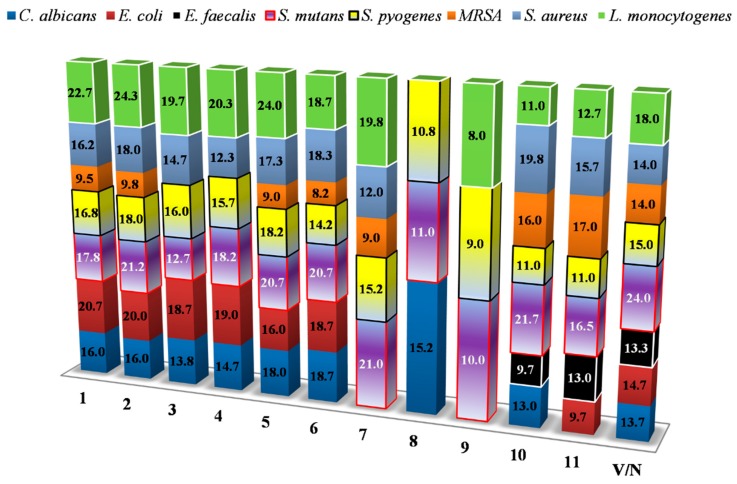
Antimicrobial potential of wood waste extracts in well-diffusion method. *V/N—Vancomycin/Nystatin. Values within columns represent a mean of inhibition zones and expressed in mm. *Q. robur* (no. 1–3), *Q. petraea* (no. 4–6), *Q. cerris* (no. 7), *Robinia pseudoacacia* (no. 8), *Prunus cerasifera* (no. 9), *Prunus avium* (no. 10), mulberry (no. 11).

Antimicrobial potential of the extracts was tested against eight representative human and opportunistic pathogens. Besides clear zones of inhibition, bacteriostatic/fungistatic effect of tested extracts was also observed. Wood waste extracts in general showed the highest antimicrobial potential against *S. mutans, S. pyogenes* and *L. monocytogenes* strains in tested concentration of 1 mg/well (Figure 4). The wild cherry extract (10) inhibited the growth of *S. mutans* and *S. aureus* yielding the largest zones of inhibition (21.7 and 19.8, respectively), compared to other extracts, towards to the mentioned pathogens. Additionally, only wild cherry and mulberry extracts (10,11) showed moderate bactericidal effect against *E. faecalis*. This indicator strain due to its higher resistance was excluded for further MIC testing. Mentioned extracts also showed high bacteriostatic effect against MRSA. Additionally, the wild cherry extract showed clear bactericidal effect only against *C. albicans* and *L. monocytogenes*, while other extracts acted more bacteriostatically. On the other hand, other wood extracts showed overwhelmingly bacteriostatic/fungistatic effect against almost all pathogens, including *E. coli*. All pathogens were susceptible to tested vancomycin and nystatin mycotic.

### 3.5. MIC Assay

For evaluation of new antimicrobials, the assessment of minimum inhibitory concentration (MIC) is usually the first step [27]. The MIC is the minimum concentration that causes visible inhibition of bacterial growth. Plant extracts with MIC < 100 µg mL^−1^ and purified compounds with MIC < 10 µg mL^−1^ are considered promising [27]. However, bactericidal activity, determined by MBC value in time-kill assays, is also an important parameter in assessing the antimicrobial activity. MBC and MIC parameters complement each other, and MBC below four times MIC value suggests the bactericidal action of a tested compound [27].

The obtained MIC values were in range from 0.02 mg mL^−1^ of extract 11, to MRSA to 2 mg mL^−1^ in the case of activity of extract 9 (myrobalan plum) against *C. albicans* (Table 3). 

The lowest MIC values (viz., 0.03 mg mL^−1^) were recorded against MRSA (extracts 5, 7 and 8), *S. aureus* (extracts 4–7) and *S. pyogenes* (extracts 1, 3, 5, 9–11). *S. mutans* also showed high sensitivity to some of the tested extracts with MICs below 0.2 mg mL^−1^. MIC values for *L. monocytogenes* were in range from 0.03–0.75 mg mL^−1^, while extracts 9–11 significantly inhibited the growth rate of this pathogen. Compared to Gram-positive isolates, *E. coli* was less sensitive to the tested extracts. *Candida albicans* showed poor sensitivity to the action of all extracts, with the exception of extract 10 with obtained MIC value of 0.25 mg mL^−1^. Alañón et al. [17] also concluded that yeasts had a stronger resistance to wood extracts than bacteria, since only toasted American oak wood and wild cherry wood extracts inhibited their growth. MICs for vancomycin, streptomycin and nystatin were lower compared to the tested extracts (0.001–0.4 mg mL^−1^). Additionally, MRSA showed resistance to all antibiotics on the highest concentration tested (0.4 mg mL^−1^). Interestingly, non-seasoned sessile oak (sample 5) showed lower MIC against MRSA and *L. monocytogenes* than seasoned oaks (samples 1, 2, 3, 4, 6).Comparing the results for *Q. robur* with the results for oak bark (*Q. robur*) [24], higher values for MIC were found against *L. monocytogenes* and *E. coli*, but lower values against *S. aureus*. In addition, the values of MIC for streptomycin were significantly lower than Elansary et al. [24] obtained. MBC and MFC values of tested extracts varied from 0.03–2 mg mL^−1^. The lowest MBC was recorded against *S. aureus* for extracts 2, 5, 8, 10 and 11. 

There was a strong simultaneous activity against all pathogens tested of extract 10 from the wild cherry wood. This could be explained by its richness of phenolic compounds which was observed in previous research [6]. For example, kaempferol is a potential candidate against different pathogenic microbes, and effective against fluconazole-resistant *Candida albicans* and Methicillin-resistant *S. aureus* (MRSA) [22]. In addition, galangin exhibited selective anti-cytochrome and antifungal activity [25], and showed antimicrobial activity against *S. aureus* [25,32], and methicillin-sensitive and methicillin-resistant *S. aureus*, *Enterococcus* spp., and *P. aeruginosa* [33]. Flavone apigenin showed strong activity against Gram-negative bacteria [34], while quercetin and apigenin derivatives showed strong antibacterial properties against Gram-negative and Gram-positive bacteria [35]. Some phenolic acids (gallic, caffeic and ferulic acids) showed antibacterial activity against Gram-positive (*S. aureus* and *Listeria monocytogenes*) and Gram-negative bacteria (*E. coli* and *P. aeruginosa*) with a greater efficiency than conventional antibiotics such as gentamicin and streptomycin [36]. Contrarily, chlorogenic acid, which was not abundant in wood species, showed no activity against Gram-positive bacteria [36]. Interestingly, noticeable antimicrobial activity of cherry wood against wine organisms was observed before [17], but, to our knowledge, its antimicrobial activity against human and opportunistic pathogens has not been investigated so far.

However, MIC values for extracts 1–7 against *S. aureus*, *L. monocytogenes* and *E. coli* were similar to MICs obtained from some other *Quercus* spp. bark extracts (Table 4), but MICs recorded towards *C. albicans* were lower compared to the results of this study. 

On the other hand, *P. avium* stem bark extracts from Nigeria showed lower antimicrobial activity against *S. aureus* and *E coli*, with MICs 6.25 mg mL^−1^ and 12.5 mg mL^−1^, respectively [43]. Compared to extracts 9 and 10, *Prunus cerasoides* showed similar antibacterial activity towards MRSA [44]. Unlike extract 11, originating from *M. alba*, bark extracts originating from *Morus mesozygia* showed significant antimicrobial activity against *C. albicans*, with obtained MIC of 0.16 mg mL^−1^ (Table 4). Interestingly, higher susceptibility of *C. albicans* was also observed for *Picea abies* and *Larix decidua* bark extracts [37]. In the literature, no significant correlation was found between antimicrobial activity and total phenolic content [17,46], as well as between antimicrobial activities and antioxidant capacity. However, structure-function of the phenolic extracts have more influence on the antimicrobial activity than the total phenol content [17]. Finally, according to Cowan [47], a wide variety of specialized metabolites show antimicrobial activity *in vitro*, such as tannins, terpenoids and alkaloids, also found in wood.

## 4. Conclusions

Wood waste from forest trees is a source of different bioactive metabolites which could find application in the food and pharmaceutical industries. Radical scavenging and antimicrobial activities of the wood waste extracts appeared to be a valuable bio-functional source. In general, HPTLC fingerprint identify the main phenolic acids present in investigated samples and revealed chemical patterns among investigated wood extracts. DPPH-HPTLC assay identified gallic, ferulic and/or caffeic acids as compounds with the highest contribution to total radical scavenging activity. Based on PCA plot, six *Quercus* samples were separated from other extracts showing strong radical scavenging activities.

Wood samples were the most active against MRSA, *S. aureus* and *S. pyogenes*. The lowest MIC and MBC values were detected in mulberry extract against MRSA. Activities were also distinguished against MRSA (extracts of non-seasoned sessile oak (5), Turkey oak, black locust and mulberry) and S*. aureus* (Turkey oak and all sessile oak extracts). The largest zones of inhibition of the growth of *S. mutans* and *S. aureus* were observed for wild cherry extract. Among sessile and pedunculate oak extracts, non-seasoned sessile oak extract (5) was distinguished by lower MIC against MRSA and *L. monocytogenes*. Extracts of myrobalan plum, wild cherry and mulberry significantly inhibited the growth rate of *L. monocytogenes*. *E. coli* was less sensitive to the tested extracts. *C. albicans* showed poor sensitivity to the action of all extracts, with the exception of the wild cherry extract.

Wild cherry wood extract can be commercially important due to good simultaneous activity against all pathogens, and is a valuable source for various formulations: Wild cherry and mulberry wood extracts with given antimicrobial activities can be especially useful in preserving perishable foods with short shelf life.

## Figures and Tables

**Table 1:** Selected wood waste extracts of different forest trees for DPPH-HPTLC (high-performance thin-layer chromatography) and antimicrobial testing assay.

Sample No.	Tree	Geographical Origin	Extraction Yield (%)
1	Pedunculate oak—*Quercus robur* L.	Slavonija (Croatia)	4.44
2		Gornji Radan (Serbia)	4.40
3		Olovo (Bosnia and Herzegovina)	4.12
4	Sessile oak—*Quercus petraea* (Matt.) Liebl.	Kučaj (Serbia)	5.06
5		Kuršumlija (Serbia)	3.05
6		Ravna Gora (Serbia)	4.58
7	Turkey oak—*Quercus cerris* L.	Kuršumlija (Serbia)	1.63
8	Black locust—*Robinia pseudoacacia* L.	Kraljevo (Serbia)	6.37
9	Myrobalan plum—*Prunus cerasifera* Ehrh	Vrnjačka Banja (Serbia)	5.80
10	Wild cherry—*Prunus avium* L.	Ravna Gora (Serbia)	3.15
11	Mulberry—*Morus alba* L.	Vrnjačka Banja (Serbia)	7.29

**Table 2:** Indicator strains used in testing antimicrobial activity of selected extracts from forest trees.

Indicator Strains	Isolate Code	Growth Medium	Growth Temperature	The Origin of The Isolates
*Streptococcus mutans*	IBR S0001	LA	37 °C	Oral cavity *
*Streptococcus pyogenes*	IBR S0004	ǁ		
Methicillin-resistant *Staphylococcus aureus* (MRSA)	ATCC33591	ǁ		Reference strains
*Staphylococcus aureus*	ATCC25923	ǁ		
*Escherichia coli*	ATCC25922	ǁ		
*Enterococcus faecalis*	ATCC29212	ǁ		
*Listeria monocytogenes*	ATCC19111	BHA		
*Candida albicans*	ATCC10231	TSA		

* Strains isolated from the human oral cavity [29]. All reference strains belong to Department of Microbiology, Faculty of Biology, University of Belgrade.

**Table 3:** Minimum inhibitory, minimum bactericidal and minimum fungicidal concentrations (MIC/MBC/MFC) of 11 wood extracts towards selected human pathogens (mg mL^−1^).

Indicator Strains/MIC (mg mL^−1^)	1	2	3	4	5	6	7	8	9	10	11	Str	Van	Nys
*S. mutans*	0.25	0.13	0.25	0.25	0.25	0.25	0.13	0.25	1.00	0.05	0.13	0.020	0.006	NT
*S. pyogenes*	0.03	0.13	0.03	0.05	0.03	0.13	0.08	0.05	0.03	0.03	0.03	0.002	0.001	NT
*S. aureus*	0.08	0.05	0.05	0.03	0.03	0.03	0.03	0.09	0.06	0.05	0.09	0.009	0.002	NT
MRSA	0.06	0.06	0.13	0.06	0.03	0.05	0.03	0.03	0.13	0.13	0.02	-	-	NT
*L. monocytogenes*	0.50	0.50	0.75	0.75	0.19	0.63	0.50	0.13	0.06	0.06	0.03	0.019	0.002	NT
*E. coli*	0.75	0.75	1.50	1.50	0.75	1.50	-	0.75	-	0.75	0.75	0.009	0.200	NT
*C. albicans*	-	-	-	-	-	-	-	-	2.00	0.25	-	NT	NT	0.006
**Indicator strains/MBC (mg mL^−1^)**	**1**	**2**	**3**	**4**	**5**	**6**	**7**	**8**	**9**	**10**	**11**	**Str**	**Van**	**Nys**
*S. mutans*	2.00	2.00	2.00	2.00	2.00	2.00	0.50	2.00	2.00	0.06	0.50	0.050	0.150	NT
*S. pyogenes*	0.50	0.50	1.00	1.00	0.50	0.50	1.00	0.25	2.00	0.50	1.00	0.050	0.050	NT
*S. aureus*	0.25	0.13	0.25	0.25	0.13	0.25	0.50	0.13	0.25	0.13	0.13	0.025	0.003	NT
MRSA	0.50	0.50	0.50	0.50	0.25	0.50	0.63	0.25	1.00	0.25	0.03	-	-	NT
*L. monocytogenes*	1.00	1.00	1.00	1.00	1.00	1.00	1.00	0.50	1.00	0.13	0.25	0.025	0.006	NT
*E. coli*	1.00	1.00	2.00	2.00	1.00	2.00	-	1.00	-	1.00	1.00	0.013	0.400	NT
*C. albicans*	-	-	-	-	-	-	-	-	-	0.50	-	NT	NT	0.025

Str—Streptomycin; Van—Vancomycin; Nys—Nystatin; NT—not tested; (-)—not determined. Values are marked with lowest value (blue), middle value (yellow), and the highest value (red). *Q. robur* (no. 1–3), *Q. petraea* (no. 4–6), *Q. cerris* (no. 7), *Robinia pseudoacacia* (no. 8), *Prunus cerasifera* (no. 9), *Prunus avium* (no. 10), mulberry (no. 11).

**Table 4:** Summarized MICs values for other waste extracts obtained from literature data.

Bark Extracts Origin MIC (mg mL^−1^)	*Sa*	Mr	*Lm*	*Sm*	*Sp*	*Ec*	*Ca*	References
*Picea abies*	0.13	-	0.16	-	-	0.08	0.97	[37]
*Larix decidua*	0.21	-	0.15	-	-	0.33	0.60	
*Quercus acutissima*	0.23	-	0.27	-	-	0.17	0.40	[24]
*Quercus macrocarpa*	0.22	-	0.29	-	-	0.13	0.34	
*Quercus robur*	0.23	-	0.25	-	-	0.10	0.31	
*Quercus robur*	0.08	-	-	-	-	0.08	-	[38]
*Quercus ilex*	0.13	-	-	-	0.51	0.26	-	[39]
*Quercus infectoria*	-	1.25	-	-	-	-	-	[40]
*Maclura tinctoria*	-	-	-	0.08	-	-	-	[41]
*Prunus africana*	0.07	0.16	-	-	-	-	-	[42]
*Prunus avium*	6.25	-	-	-	-	12.50	-	[43]
*Prunus cerasoides*	5.00	1.00	-	-	-	-	1.00	[44]
*Morus mesozygia*	0.16	-	-	-	-	0.04	0.16	[45]

*Sa*—*S. aureus*; Mr—MRSA; *Lm*—*L. monocytogenes*; *Sm*—*S. mutans*; *Sp*—*S. pyogenes*; *Ec*—*E. coli*; *Ca*—*C. albicans*; (-)—not tested.
